# Stage-dependent trade-offs in thermal performance: fluctuating temperatures reverse larval and adult fitness in *Anopheles gambiae* and *An. coluzzii*

**DOI:** 10.1093/jme/tjaf189

**Published:** 2026-01-30

**Authors:** Mauro Pazmiño-Betancourth, Alena Miller, Maria Katsoni, Ivan Casas Gomez-Uribarri, Fredros O Okumu, Simon A Babayan, Francesco Baldini

**Affiliations:** School of Biodiversity, One Health and Veterinary Medicine, University of Glasgow, Glasgow, UK; School of Biodiversity, One Health and Veterinary Medicine, University of Glasgow, Glasgow, UK; School of Biodiversity, One Health and Veterinary Medicine, University of Glasgow, Glasgow, UK; School of Biodiversity, One Health and Veterinary Medicine, University of Glasgow, Glasgow, UK; School of Biodiversity, One Health and Veterinary Medicine, University of Glasgow, Glasgow, UK; Department of Environmental Health and Ecological Sciences, Ifakara Health Institute, Ifakara, Tanzania; School of Biodiversity, One Health and Veterinary Medicine, University of Glasgow, Glasgow, UK; School of Biodiversity, One Health and Veterinary Medicine, University of Glasgow, Glasgow, UK; Department of Environmental Health and Ecological Sciences, Ifakara Health Institute, Ifakara, Tanzania

**Keywords:** Anopheles gambiae s.s, Survival, Life History, diurnal temperature fluctuation, fitness

## Abstract

Temperature is a key environmental factor influencing the development, survival, and transmission potential of malaria vectors. While most laboratory studies use constant temperature (CT) regimes, mosquitoes in natural habitats experience fluctuating temperatures (FTs), which may affect their life-history traits. We investigated the effects of CT (27 °C) and FT (27 ± 3 °C) on larval and adult traits of 2 major malaria vectors, *Anopheles gambiae* and *An. coluzzii*, under laboratory conditions. We measured larval survival, development time, adult body size, and adult survival, using survival and mixed-effects models. Species-specific and stage-specific responses to temperature regimes were observed. *An. gambiae* larvae exhibited higher survival under FT, while *An. coluzzii* larvae survived better under CTs. However, this pattern reversed in adulthood: *An. coluzzii* adults showed increased survival and larger body size under FT, whereas *An. gambiae* adults performed better under CT. Development time was slightly longer under FT for both species, with *An. coluzzii* pupating faster overall. These opposing patterns suggest that differential larval survival under FTs may influence adult fitness in a species-specific manner. The contrasting and reversed responses of *An. gambiae* and *An. coluzzii* across life stages might reflect their ecological adaptations: *An. gambiae*, found in small and thermally variable habitats, performed better under FT during larval stages, while *An. coluzzii*, associated with larger, more thermally stable habitats, showed improved adult performance under FT. These findings underscore the importance of incorporating species-specific, stage-dependent thermal responses into models of vector dynamics and control strategies under climate change.

## Introduction

Climate change—including increased global temperatures (Climate Action Tracker 2023) and the rising frequency of extreme weather events (Stocker 2014, Fischer and Knutti 2015)—is predicted to impact the ecology of mosquitoes and their ability to transmit malaria and other vector-borne diseases (VBDs) (Thomson and Stanberry 2022). Indeed, the life-history traits of mosquitoes, including the major malaria vectors *Anopheles gambiae*, *An. coluzzii*, *An. arabiensis*, and *An. funestus*, are heavily influenced by environmental factors such as temperature, humidity, and rainfall (Afrane et al. 2012, Parham et al. 2012, Charlwood 2019). As ectotherms, mosquitoes are highly sensitive to temperature, which influences both their aquatic and terrestrial life stages (Caminade et al. 2019, Villena et al. 2022), as well as the development of the malaria parasite within the vector (Paaijmans et al. 2013a, Suh et al. 2024). As global warming is altering not only average daily temperatures but also the amplitude and frequency of temperature fluctuations (Colinet et al. 2015), understanding how fluctuating temperatures (FTs) affect mosquito ecology and malaria transmission dynamics under future climate change scenarios is vital [28]. This is also critical for informing climate-resilient vector control interventions (Ferguson and Govella 2023) and to optimize mosquito rearing protocols for sterile insect techniques (SIT), *Wolbachia*-based approaches, and gene drive technologies (Wang et al. 2025). Indeed, while most laboratory studies rear mosquitoes under constant temperature (CT) conditions (Christiansen-Jucht et al. 2014, Agyekum et al. 2021, Barr et al. 2023, 2024, Martin and Hillyer 2024), data on the effects of FT on mosquito ecology remain limited (Lyons et al. 2013, Paaijmans et al. 2013b, Davies et al. 2016, Hidalgo et al. 2016, 2018, Mamai et al. 2016).

In ectotherms, the association between temperature and life-history traits such as development, survival, and reproduction—commonly described through thermal performance curves (TPCs)—typically follows a unimodal function: performance increases from zero at a critical minimum (CT_min_), peaks at an optimum (T_opt_), and declines to zero at a critical maximum (CT_max_) temperature. While T_opt_ represents the temperature at which metabolic performance is maximized, CT_min_ and CT_max_ mark physiological thresholds beyond which cellular functions are severely impaired due to protein denaturation, disrupted membrane fluidity, mitochondrial dysfunction, and oxidative stress (Duffy et al. 2015, Bodlah et al. 2023). In mosquitoes, TPCs have been consistently quantified for traits including larval and adult survival, development rate, biting frequency, and reproductive output, highlighting performance constraints at both thermal extremes (Mordecai et al. 2013, Johnson et al. 2015, Villena et al. 2022).

Typically, TPCs exhibit an accelerating temperature–performance relationship below T_opt_ and a decelerating relationship above T_opt_, as shown in early work by Kaufmann (Kaufmann 1932) and known as Jensen’s inequality (Jensen 1906). This framework helps explain discrepancies between insect performance under CTs and FTs with the same mean. Specifically, when FT occurs in the accelerating portion of the curve (below T_opt_), performance often exceeds that observed under CT; conversely, when FT occurs above T_opt_, performance tends to be reduced compared to CT (Colinet et al. 2015). The magnitude and direction of these effects depend on 2 key factors: the thermal sensitivity of the trait in question (i.e., how steep or curved the TPC is), and the amplitude of temperature fluctuations. Traits with greater thermal sensitivity (steeper TPCs) and environments with larger thermal amplitudes exhibit stronger deviations between FT and CT outcomes (Colinet et al. 2015). Global warming is expected to increase the occurrence of these deviations. Since thermal sensitivity likely varies among mosquito species, current models of mosquito development may underestimate the significance of FT.

Mosquitoes undergo larval development in aquatic habitats that often experience marked diurnal temperature fluctuations. In sub-Saharan Africa, the primary malaria vectors exhibit species-specific habitat preferences. For instance, *Anopheles funestus* tends to occupy larger, deeper, and more stable water bodies (Kahamba et al. 2024), whereas members of the *An. gambiae* sensu lato complex typically breed in shallow, sunlit, and often ephemeral pools (Onen et al. 2021). Within this complex, *An. gambiae* and *An. coluzzii* differ in their geographic distributions, ecological preferences, and roles in malaria transmission (Wiebe et al. 2017, Mbama Ntabi et al. 2024). *An. coluzzii* predominates in savannah regions, particularly in southern West Africa, where it breeds in more permanent and human-modified habitats such as rice fields (Cassone et al. 2014). In contrast, *An. gambiae* is more common in forested areas, primarily in northern and central regions, and prefers temporary, rain-fed water bodies such as puddle margins (Gimonneau et al. 2010, Mattah et al. 2017, Koukpo et al. 2019). Although these ecological differences influence the seasonal dynamics of malaria transmission—*An. coluzzii* being more prevalent during the dry season and *An. gambiae* during the rainy season (Akogbéto et al. 2018)—the extent to which their distinct ecologies affect thermal sensitivity remains unknown.

The adaptation of *An. gambiae* and *An. coluzzii* to different habitats suggests that their thermal sensitivity and experienced thermal fluctuations may be different. However, while some data are available for *An. gambiae* and other *Anopheles* species, information on *An. coluzzii* is scarce. Most studies in laboratory conditions have relied on CT, with few using FT (Lyons et al. 2013, Paaijmans et al. 2013b, Davies et al. 2016, Hidalgo et al. 2016, 2018, Mamai et al. 2016, Shocket et al. 2025). Studies on life-history traits of *An. gambiae* using CT have shown that larval development is faster at high temperatures (Bayoh and Lindsay 2003), while both body size and survival decrease (Agyekum et al. 2021, 2022, Barr et al. 2023). Similar trends have been found in *An. coluzzii* with added interaction of survival with humidity (Faiman et al. 2017). The studies using FT have shown that *An. coluzzii* can adjust its cuticular hydrocarbons, increase its metabolic rate, and decrease its protein content when reared in low-temperature, low-humidity conditions, while *An. gambiae* does not (Hidalgo et al. 2016, 2018). However, life history and survival studies with extended follow-up times in both species are lacking. In other *Anopheles* species, it has been shown that FTs that include CT_min_ and CT_max_ values (18 to 35 °C) increase the survival of *An. arabiensis* larvae when reared in a mix with *An. quadriannulatus* (Davies et al. 2016). FT has a minimal effect on the survival of adult *An. arabiensis* when fluctuations are large (±20 °C) with a maximum temperature of 35 °C, compare to *An. funestus* whose survival greatly decreases even with small fluctuations (±10 °C) with a maximum temperature of 30 °C (Lyons et al. 2013). Overall, the literature collectively indicates that FTs alter mosquito life-history responses relative to constant conditions. Since mosquitoes are naturally exposed to FT conditions, there is a need to expand our understanding of these effects on vectors relevant for malaria transmission (Pascual et al. 2009, Paaijmans et al. 2010, 2013b, Shocket et al. 2025).

In this study, we compared the effects of CT and FT temperatures, both with a mean of 27 °C (the standard laboratory temperature for mosquito rearing), on key larval and adult life-history traits in *An. gambiae* and *An. coluzzii* in controlled laboratory settings. Our findings reveal contrasting species-specific responses across life stages. *An. gambiae* exhibited improved larval performance under FT relative to CT, whereas *An. coluzzii* performed better under CT. In adulthood, however, the pattern reversed: *An. coluzzii* showed increased survival and body size under FT, while *An. gambiae* fared better under CT. We hypothesized that *An. gambiae*, adapted to ephemeral, thermally variable habitats, would perform better under FTs, while *An. coluzzii*, associated with more stable aquatic environments, would benefit from constant conditions, though these patterns may vary across life stages.

## Materials and Methods

### Temperature Design

We assessed the effect of 2 temperature regimes on life-history traits of *An. gambiae* and *An. coluzzii* using environmental incubators (CLF Plant Climatics). The 2 temperature regimes were 27 °C (CT) and 27 ± 3 °C (FT), with daily temperature range (DTR) fluctuating between 24 °C and 30 °C, peaking at 12:00 pm, based on typical diurnal patterns in sub-Saharan Africa (Charlwood 2017, Suh et al. 2020), Supplementary Fig. S1). The shape of temperature fluctuations was programmed manually to be approximately sinusoidal. The rate of change between the different programmed points was linear. The 2 regimes had the same mean temperature; thus, any changes in life-history traits between the two would depend on the presence/absence of the fluctuation. We programmed a photoperiod of 12:12 h (L:D) cycle, with alternations occurring at 8:00 AM and 8:00 PM, and relavtive humidity (RH) was programmed at 75 ± 10% (as per the chamber’s specifications) across all experiments. This was constantly monitored using independent data loggers, which confirmed that the value always stayed within range.

### Larval Rearing, Temperature Regimes, and Feeding

Two strains were used in the study: *Anopheles gambiae* Kisumu strain originating from Kisumu, Kenya, and *Anopheles coluzzii*, Ngousso strain from Yaoundé, Cameroon (Harris et al. 2010). For each replicate and species, 10 trays with 200 larvae and 500 ml of deionized water were placed into each environmental chamber. Larvae were fed with Tetramin Tropical Flakes and Tetra Pond Pellets (Tetra Ltd., United Kingdom) at approximately 0.92 mg/larva/day and reared into adults. For body size assessment, pupae were collected and placed in emergence cages. To control for the effects of larval development rate on adult size, each emergence cage contained the same number of pupae from larvae with fast, normal, and slow development rates. This was achieved by combining pupae collected during early (8 to 10 d), peak (11 to 14 d), and late (15 to 16 d) pupation times in each cage (*n* = 10 pupae per time point). For adult survival analysis, a total of 400 pupae were put into each cage and reared under the same conditions as they were reared as larvae. The day the pupae emerged into adults was assigned as day 0. Adults were fed with 5% glucose solution *ad libitum*. Females were blood-fed human blood through a membrane feeder 3 to 4 d after emergence and every 3 to 4 d subsequently. Three independent experimental blocks were conducted over consecutive weeks.

### Adult Survival and Body Size

Adult survival was monitored for 20 d. Dead mosquitoes were collected, sexed, and counted daily. Wing lengths (distance from the alula notch to the tip) of dead males and females were measured using DinoCapture 2.0, under a Leica MZ95 microscope, and used as a proxy for body size (Maïga et al. 2012). Observers were not blinded.

### Statistical Analysis

Analyses were carried out using R version 4.4.0 (R Core Team 2021), as follows: (1) For larval development time to pupation (L1 to pupation), a linear mixed model (LMM) was run using the “lmer” function from the “lme4” (Bates et al. 2015) package in R. Day of pupation was used as the response variable, temperature regime, species and their interaction were the explanatory variables with replicate as random effect. (2) For larval survival (measured as the proportion of L1 larvae that developed into pupae), a generalized linear mixed model (GLMM) with binomial distribution was run using the “glmer” function. Larval survival was the response variable, temperature regime, species, and their interaction were the explanatory variables, and replicate was the random effect. (3) To examine the effect of temperature on body size, an LMM was run using the “lmer” function. Wing length was used as the response variable. Temperature regime, species, and their interaction were used as explanatory variables; replicate was treated as a random effect.

Survival data were analyzed using Cox’s proportional hazards and parametric models using the package “survival” (Therneau and Grambsch 2000) and “flexsurvreg” (Jackson 2016). Proportional hazard assumptions were tested using the function cox.zph from the survival package. For parametric survival, the Akaike information criterion (AIC) was used to choose the best-fit distribution (Supplementary Table S1). A random intercept for replicate was included using a frailty term in the Cox model. Explanatory variables were species (2 levels), sex, and temperature regime (CT or FT), and the interaction between temperature and species. A separate Cox’s proportional hazards model was developed for females only, incorporating wing length as an additional explanatory variable. The significance of each variable was calculated through stepwise model simplification based on likelihood ratio tests using the “lmerTest” package for all models. Model assumptions were tested using the “DHARMa” package (Hartig 2024). Residuals showed no evidence of overdispersion or spatial/temporal autocorrelation.

## Results

### Larval Survival and Development Time

**Fig. 1.**
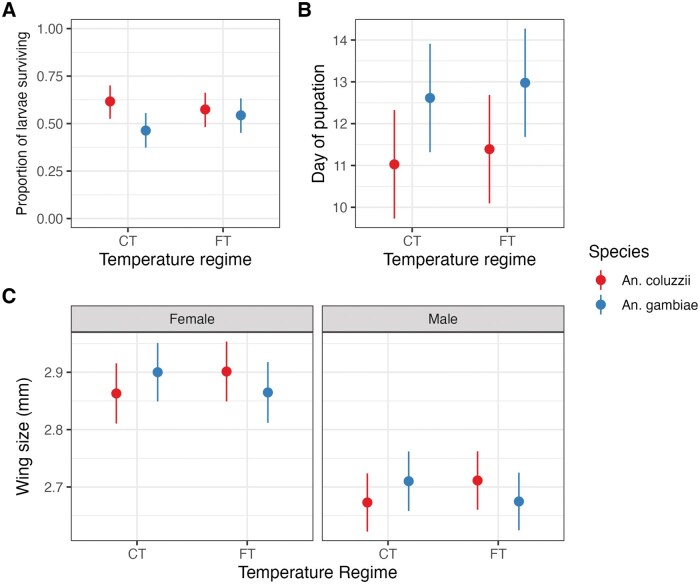
Influence of temperature and species on larval survival, developmental time, and body size. (A) Predicted average number of larvae that survived stratified by species and different temperature conditions. Points represent the mean, and the lines represent the 95% confidence intervals. (B) Day of pupation by species and temperature regime. (C) Predicted wing sizes of *An. gambiae* (blue) and *An. coluzzii* (red) are divided into females and males under constant and fluctuating temperature conditions.

*An. coluzzii* larvae survival was higher at CT than at FT, whereas *An. gambiae* survival was higher under FT (interaction between species and temperature χ^2^ = 16.24, *P* < 0.001; Fig. 1A, Table 1).

**Table 1.** Statistics for the model on association temperature and life traits

Type of model	Response variable	Explanatory variables	χ^2^	*P*-value
**GLMM**	Larval survival	Temperature × Species	16.24	<0.001
**LMM**	Development time	Temperature × Species	3.15	0.07
		Temperature	20.85	<0.001
		Species	361.48	<0.001
**LMM**	Wing length (main effect)^a^	Sex	124.51	<0.001
		Temperature × Species	5.85	<0.01

Interaction between explanatory variables is depicted by the symbol (×).

a Wing length is measured in millimeters.

Conversely, while there was no interaction between temperature regime and species on developmental time, both variables were associated with the time that L1 larvae took to reach the pupal stage (Species: χ^2^ = 361.48, *P* < 0.001, temperature regime: χ^2^ = 20.85, *P* < 0.001) (Table 1), but the interaction was not significant. Unexpectedly, the developmental time in *An. coluzzii—*predicted to be a slower pace species—was, in fact, slightly shorter under both temperature regimes (Fig. 1B). *Anopheles gambiae* larvae took 1.58 d longer to pupate than *An. coluzzii* (95% CI: 1.43 to 1.74, *P* < 0.001). Overall pupation for both species was slightly faster at CT, as larvae under FT required an extra 0.36 d (95% CI: 0.21 to 0.52) to pupate.

### Body Size

To understand if the temperature regime influenced pupal growth, the wing length of adults emerging from pupae collected from different days was measured as a proxy of their body size. There was a strong association between wing length and sex (χ^2^ = 124.51, *P* < 0.001), and a significant interaction between temperature and species (χ^2^ = 5.84, *P* < 0.01; Table 1). Specifically, *An. coluzzii* were larger when reared under FT than CT, whereas *An. gambiae* showed the opposite pattern (Fig. 1C).

### Adult Survival

**Fig. 2.**
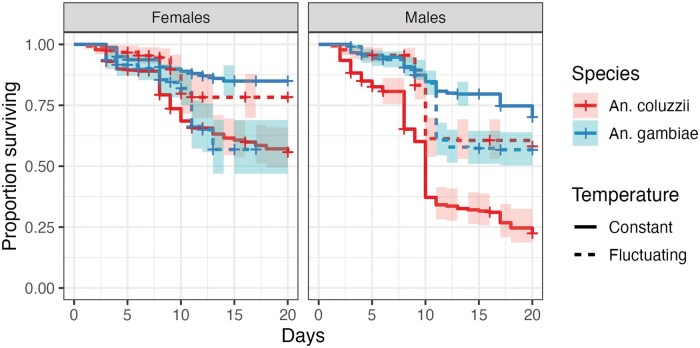
Kaplan–Meier survival plot of adult females and males, *An. gambiae* (blue line) and *An. coluzzii* (red line) reared under constant (solid line) and fluctuating temperature regimes (dashed line). Mortality of mosquitoes was recorded up to day 20. The shaded areas depict the 95% confidence intervals.

**Fig. 3.**
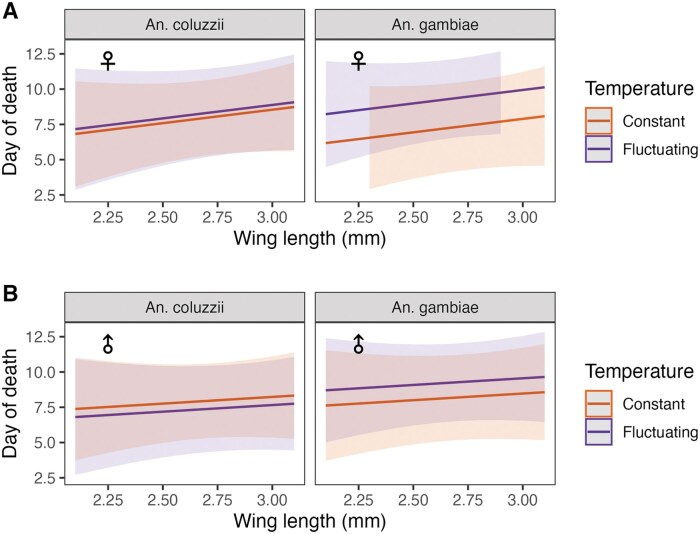
Relationship between longevity and wing length (mm) of *An. gambiae* and *An. coluzzii* at constant (orange line) and fluctuating (purple line) temperatures for females (A) and males (B). The relationship is visualized by the line of best fit (regression line), and the shaded areas represent the 95% confidence intervals.

The effect of temperature on adult survival differed significantly between the 2 species (χ^2^ = 78.32, *P* < 0.001). In contrast to the effect of temperature regime on the 2 species during larval stages, during adulthood, *An. coluzzii* live longer at FTs compared to constant ones, while the opposite was observed for *An. gambiae*. Sex also influenced survival (χ^2^ = 29.74, *P* < 0.001), as males generally survived less than females (Fig. 2). To test the effect of body size on survival, a model including only females was created. When wing length was included, the significant effect of the interaction between temperature and species was still observed (χ^2^ = 23.82, *P* < 0.001). As expected, wing length was positively associated with survival (χ^2^ = 36.19, *P* < 0.001; Table 2) as larger individuals tended to live longer (Fig. 3). This finding suggests that the effects of temperature regimes on the survival of the 2 species were independent of their body size.

**Table 2.** Results of the likelihood ratio test showing the significance of each explanatory variable for the Cox proportional hazards models on the effect of temperature, species sex, and wing length on survival

Type of model	Response variable	Explanatory variables	χ^2^	*P*-value
**Parametric survival model with log-normal distribution (full model)**	Survival	Temperature × Species	78.32	<0.001
		Sex	29.74	<0.001
**Cox proportional hazard (females only model)**	Survival	Temperature × Species	23.82	<0.001
		Wing length	36.19	<0.001

Interaction between explanatory variables is depicted by the symbol (×).

## Discussion

This study compared the effect of CT and FT on life-history traits of 2 *Anopheles* species, *An. gambiae* and *An. coluzzii*. We found that *An. gambiae* had higher larval survival and longer pupation time under FT, but larger adult body size and better adult survival under CT. In contrast, *An. coluzzii* showed higher larval survival under CT, but increased pupation time, larger adult size, and better adult survival under FT, showing species-specific thermal sensitivities across life stages.

These differences could be explained by the innate hard-wired preferences of different types of breeding sites for each species. Indeed, *An. gambiae* exploits temporary breeding sites (Hidalgo et al. 2018, Ebhodaghe et al. 2024), typically small and shallow, where temperature fluctuates more. On the contrary, *An. coluzzii* can be found in a wider variety of breeding sites with a preference to more permanent breeding sites, which are large and deep, where temperature variation is reduced (Gimonneau et al. 2012, Hidalgo et al. 2018). In contrast, *An. arabiensis* larvae show increased survival under FT (Davies et al. 2016), highlighting species-specific responses (Davies et al. 2016). However, similar to *An. gambiae*, FT increases larval survival in *An. stephensi* (Paaijmans et al. 2010). Both species pupated more slowly at FT, with an extra 0.36 d required to pupate. Similar studies on *An. arabiensis* and *An. quadriannulatus*, showed faster developmental times at CTs compared to fluctuating (Davies et al. 2016). Overall, *An. coluzzii* pupated faster across both temperature regimes compared to *An. gambiae*. This increase in fitness of *An. coluzzii* has been reported before in laboratory colonies, and it might be caused by colony-specific genetic drift (Gimonneau et al. 2014).

We found that the effect of FT on adult body size differed between species. *An. gambiae* adults reared under CT emerged larger than those reared under FT, whereas *An. coluzzii* exhibited the opposite pattern, with larger adults emerging under FT. These differences may reflect species-specific responses in larval survival. In *An. gambiae*, higher larval mortality under CT could have led to reduced competition for resources among the surviving individuals, enabling greater growth. Conversely, in *An. coluzzii*, improved larval survival under CT may have resulted in increased intraspecific competition, resulting in smaller emerging adults (Jannat and Roitberg 2013). Furthermore, differences in larval survival between the 2 species may have led to variations in bacterial proliferation and the accumulation of toxic waste products, which could have subsequently influenced larval development. The differences in wing lengths are the opposite of what was observed for larval survival, showing that when larval survival is higher for 1 species in 1 temperature regime, their body size is reduced, suggesting that larval density and competition—higher if more larvae survive—impacted on adult body size. While larval density was not directly manipulated, differences in survival may have led to varying levels of intraspecific competition, potentially influencing adult size.

The conditions experienced during the larval stage might have influenced also adult-stage outcomes. While *An. gambiae* adults showed higher survival under CT, *An. coluzzii* adults survived better under FT—a reversal of the larval survival patterns observed. This suggests that reduced larval competition, resulting from higher mortality (i.e., *An. gambiae* at CT and *An. coluzzii* at FT), may have conferred survival advantages to those individuals that reached adulthood. Importantly, our models included body size as an explanatory variable, indicating that these species-specific differences in adult survival are not simply mediated by body size, but may instead reflect selection processes acting during the larval stage. For example, the suboptimal temperature conditions that led to increased larval mortality may have acted as a selective filter, allowing only the most robust individuals to reach adulthood. Consequently, selective mortality during the larval stage may result in a cohort of more robust adults, potentially confounding direct temperature effects on adult survival—where the relationship between temperature and adult traits is conditioned on having survived the larval stage, rather than being a direct effect of the temperature regime itself. Moreover, the different survival to temperatures might not be fully mediated by their larval development, but instead by a different physiological response during the adult stage. The observed species differences may also be influenced by species-specific thermal performance. Since our FT conditions reached up to 30 °C—likely exceeding Topt for both species—the FT treatment could have differently reduced their performance during periods when temperatures surpassed their optimal range, depending on the shape of their TPCs.

## Conclusion

This study highlights how even small daily temperature fluctuations can influence the life-history traits of *Anopheles* mosquitoes, with distinct responses observed between species under fluctuating versus CT conditions. These findings suggest that laboratory studies using CTs may misrepresent field performance, particularly for species like *An. gambiae* that exploit thermally variable habitats. This shows the importance of including daily variations when studying the impact of temperature and other environmental conditions on mosquito ecology. Future works should include larger fluctuations in temperature as well as humidity to gain a deeper understanding of the effects of ecological factors on life-history traits.

## Supplementary Material

tjaf189_Supplementary_Data

## Data Availability

All data and code to reproduce this analysis and figures are available at https://github.com/maurocolapso/Anopheles_ConstantvsFluctuating.git.
