# Supplementary material for: Stage-dependent trade-offs in thermal performance: fluctuating temperatures reverse larval and adult fitness in Anopheles gambiae and An. coluzzii
Source: J Med Entomol. 2026 Jan 30;63(1):tjaf189. doi: 10.1093/jme/tjaf189 (PMC12861982; doi:10.1093/jme/tjaf189)
Supplement: tjaf189_Supplementary_Data [file tjaf189_supplementary_data.pdf]

Supplementary Material

Stage-Dependent Trade-Offs in Thermal Performance: Fluctuating Temperatures Reverse Larval and Adult Fitness in *Anopheles gambiae* and *An. coluzzii*

Mauro Pazmino[1,*], Alena Miller[1], Maria Katsoni[1], Ivan Casas Gomez-Uribarri[1], Fredros O Okumu[1,2], Simon A Babayan[1], Francesco Baldini[1,2,*]

[1]School of Biodiversity, One Health and Veterinary Medicine, University of Glasgow, UK, G12 8QQ

[2]Department of Environmental Health and Ecological Sciences, Ifakara Health Institute, Ifakara, Tanzania

*mauro.pazminobetancourth@glasgow.ac.uk

*francesco.baldini@glasgow.ac.uk

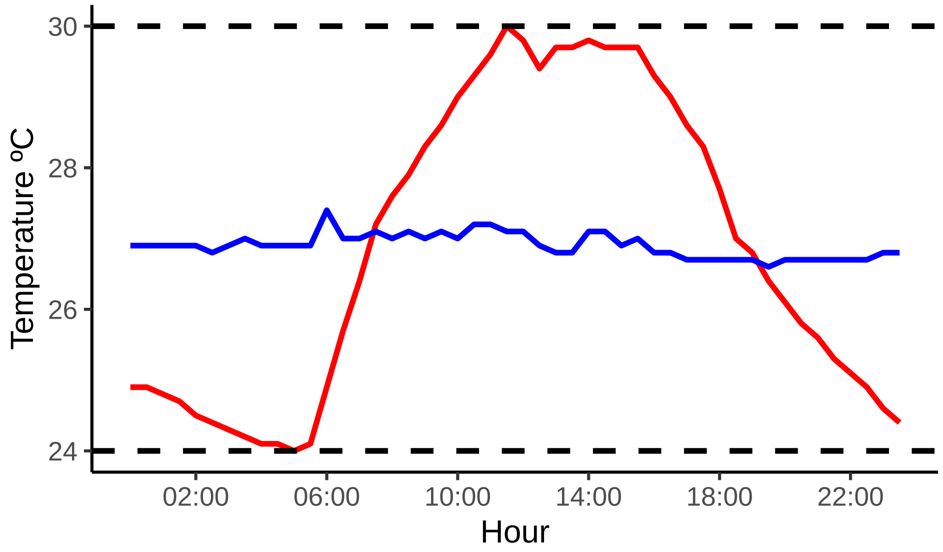

Figure S1 Example of temperature regimes used on the essays. Temperature regimes used on the essays. Blue line shows the constant 27 degrees and red line shows the fluctuations across the day with a range of 3 degrees. Data collected from a data logger each half an hour for a day.

Table S1. Akaike Information Criterion (AIC) values for different survival model fitted with survival data with different distributions. Best fit model and the AIC values are bolded.

Distribution	AIC
Weibull	4499.793
**Lnorm**	**4462.604**
Llogis	4473.795
Exponential	4606.482
Gompertz	4566.760
